# “Single-pole dual-control” competing mode in plants

**DOI:** 10.3389/fpls.2023.1149522

**Published:** 2023-06-30

**Authors:** Tian-Ying Yu, Tian-Ying Gao, Wen-Jia Li, Dan-Lu Cui

**Affiliations:** College of Life Sciences, Yantai University, Yantai, China

**Keywords:** single-pole dual-control, autocrine, paracrine, antagonism, symbiosis, immunity, iterative development

## Abstract

Plant development and pattern formation depend on diffusible signals and location cues. These developmental signals and cues activate intracellular downstream components through cell surface receptors that direct cells to adopt specific fates for optimal function and establish biological fitness. There may be a single-pole dual-control competing mode in controlling plant development and microbial infection. In plant development, paracrine signaling molecules compete with autocrine signaling molecules to bind receptors or receptor complexes, turn on antagonistic molecular mechanisms, and precisely regulate developmental processes. In the process of microbial infection, two different signaling molecules, competing receptors or receptor complexes, form their respective signaling complexes, trigger opposite signaling pathways, establish symbiosis or immunity, and achieve biological adaptation. We reviewed several “single-pole dual-control” competing modes, focusing on analyzing the competitive commonality and characterization of “single-pole dual-control” molecular mechanisms. We suggest it might be an economical protective mechanism for plants’ sequentially and iteratively programmed developmental events. This mechanism may also be a paradigm for reducing internal friction in the struggle and coexistence with microbes. It provides extraordinary insights into molecular recognition, cell-to-cell communication, and protein–protein interactions. A detailed understanding of the “single-pole dual-control” competing mode will contribute to the discovery of more receptors or antagonistic peptides, and lay the foundation for food, biofuel production, and crop improvement.

## Introduction

“Single-pole dual-throw switch (SPDT switch)” and “single-pole single-throw switch (SPST switch)” are concepts of circuits in physics. The “pole” is the controller of the switch. A single pole or double pole usually represents the number of controllers. “Throw” means the circuit to be controlled or started by the controller “pole.” Single and double throws are usually used to show how many circuits will be held. The SPST switch is a circuit controlled by a controller. Thus, the SPDT switch is a controller that can be pushed to both sides for dual control and a circuit conversion device that controls power output in two directions.

**Figure 1:**
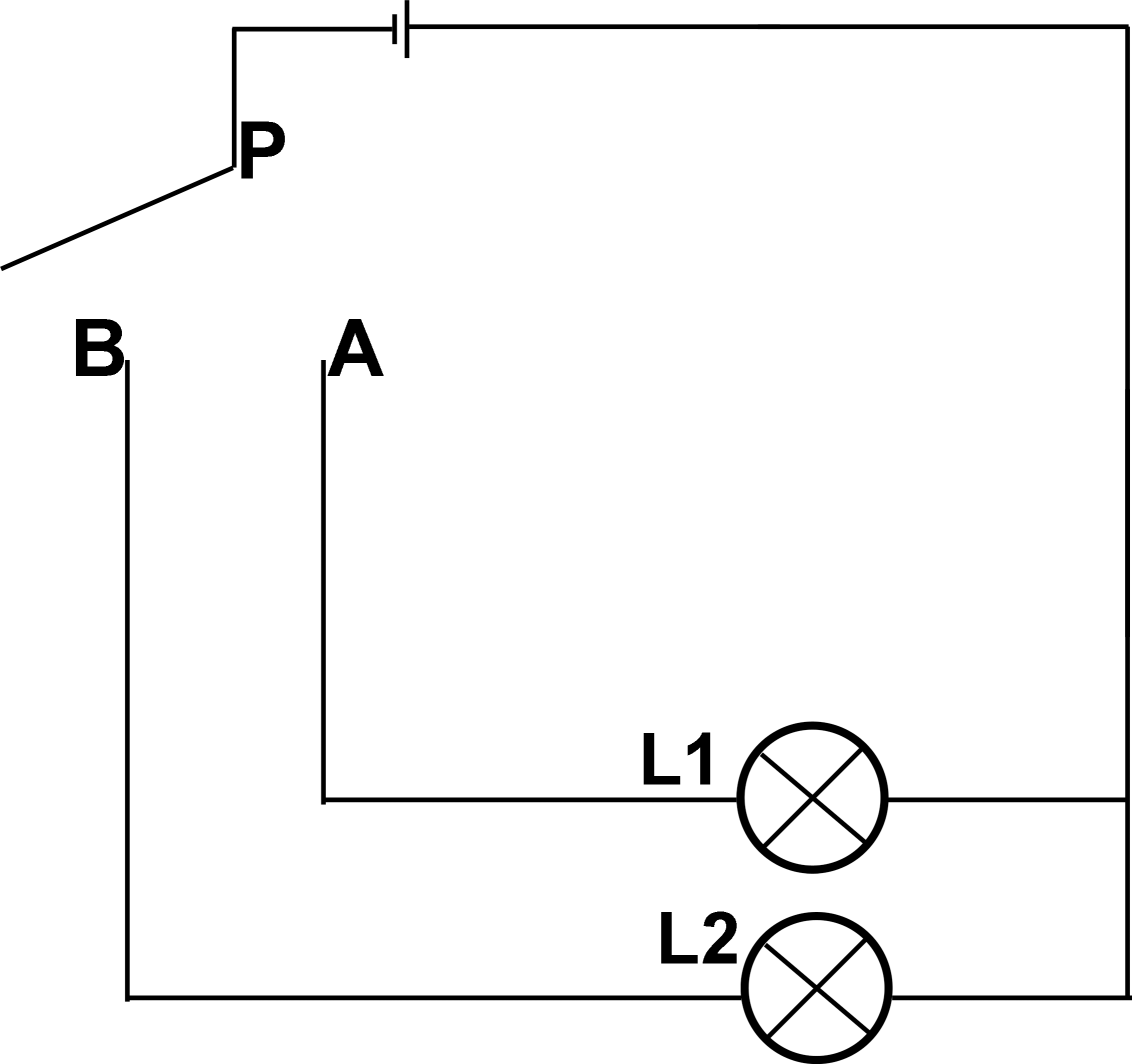
Circuit diagram of single pole double throw switch. When the movable handle P is connected to the output end **(A)**, the L1 light of circuit A is turned on, and circuit B is disrupted. In contrast, if the movable handle P is connected to **(B)**, the B circuit starts, the L2 light is on, and the A circuit is disconnected.

We used a simple circuit for illustration, as shown in **Figure 1**. The SPDT switch consists of moving and fixed ends, and the movable end is the so-called “pole.” It is the power input terminus and is connected to the handle of the switch; the other two fixed ends are the power output ends, which are associated with the electrical equipment. When the movable handle P is connected to the output end (A), the L1 light of circuit (A) is turned on, and circuit B is disrupted. In contrast, if the movable handle P is connected to B, the B circuit starts, the L2 light is on, and the A circuit is disconnected. In this way, the SPDT switch can control two devices or switch the running direction. The two controlled devices cannot be operated simultaneously, and the two circuits are in a repulsive or competitive relationship. The “pole” of the controlling switch is the competitive key to deciding which circuit will be connected. For the control switch, the two output circuits are equal.

Although a large number of peptides and receptors have been found, most of them are one-way positive signal pathways. Only a limited number of cases regulate the signal pathway in an antagonistic manner (Lee and De Smet, 2016). We proposed that the antagonistic competitive signaling pathways adopt a “single-pole dual-control (SPDC)” competing mode in plants. Here, “pole” refers to receptors or receptor complexes, and “dual control” means that different two-set peptide ligands with similar or different structures compete to bind the same receptor or receptor complex (single pole) to initiate opposite or antagonistic signaling pathways. The regulation of the SPDC competing mode is similar to the SPDT switch in the circuit. We propose that the SPDC competing mode can precisely control the maturation or germination of pollen at the appropriate spatiotemporal window in pollen development. In the interaction between pollen and stigma papilla cells, we speculate that the competition mode of SPDC finely controls the ROS homeostasis of papilla cells, which is associated with the hydration and germination of pollen. In pollen tubes transporting sperm cells, we suggest that the competing mode of SPDC regulates the integrity of pollen tube growth and the rupture of pollen tubes. In the process of stomatal patterning formation, the meristemoid mother cell’s division or the meristemoid’s differentiation might be determined through the SPDC competing mode.

We found that the programmed developmental signal in plants depends on the iterative initiation of the SPDC competing mode, which is an economical and effective strategy. It may be of universal significance for fine regulation in developmental progress. We also found that the SPDC competing mode functions in the interaction between symbiotic fungi and plants. The discovery of this SPDC competing mode could guide us to study more signal networks regulated by ligand–receptor pairs and accelerate the systematic understanding of the developmental program of plants. The molecular mechanism of the SPDC competing mode provides extraordinary insights into molecular recognition, intercellular communication, and protein–protein interactions. We illustrate this mode with limited available examples and point out how this mode might contribute to deepening the understanding of peptide signals and promoting research progress. A deep understanding of the SPDC competing mode offers new ideas for food production, crop improvement, and molecular breeding.

## Inhibition and initiation of pollen germination

The SPDC competing mode exists in the development progression of pollen. In tomato, the pollen-specific LATE ANTHER TOMATO52 (LAT52) peptide interacts with the pollen-specific receptor kinases (LePRK1/2) on the vegetative cell membrane to inhibit pollen germination before maturity (Tang et al., 2002). Once the pollen lands on the stigma, the stigma-specific LeSTIG1 peptide competes with LAT52 to interact with LePRK1/2, disconnecting the signaling pathway in the pollen to inhibit premature germination, and triggering the downstream signaling pathways of LeSTIG1-LePRK1/2 to facilitate pollen germination (Tang et al., 2004). In the SPDC competing mode, LePRK1/2 acts as the “pole,” and LAT52 and LeSTIG1 peptide ligands act as “dual control switches” to inhibit or promote pollen germination by interacting with LePRK1/2.

The expression of *LAT52* begins at the tetrad stage of anther development and gradually increases during male gametogenesis until the pollen maturity stage ends (Twell et al., 1989). An 18 KD mature LAT52 peptide is produced at the later stage of anther development and plays an essential role as a ligand signal in pollen hydration and germination (Twell et al., 1990; Muschietti et al., 1994).

The pollen receptor kinase gene *LePRK2* is expressed in mature pollen and significantly increases in pollen tubes. Tang et al. (2002) detected the complexes of LAT52-LePRK2 in mature pollen and found only LePRK2 in pollen tubes (Tang et al., 2002). It suggested that the interaction of LAT52 and LePRK2 dissociates with pollen germination (Tang et al., 2002). Pollen-specific LAT52 binds to the receptor LePRK2 on the pollen surface, enabling pollen to monitor itself and prevent it from germinating before maturity and pollination. LAT52-LePRK2 inhibited pollen pre-pollination germination and became part of the SPDC competing mode.

The tomato stigma-specific LeSTIG1 is a small cysteine-rich 8 to 10 KD protein; it binds to the extracellular domain of LePRK2 *in vitro*. Exogenous application of LeSTIG1 promoted pollen tube growth *in vitro*. The LePRK1-LePRK2 complexes and LeSTIG1 were found in stigma and style extracts (Wengier et al., 2003). These findings suggest that stigma-specific LeSTIG1 interacts with pollen-specific LePRK1/2 to promote pollen germination and tube growth in the stigma or style (Tang et al., 2004). The intracellular components regulated by LeSTIG1-LePRK1/2 are unknown. The signaling pathway regulated by LeSTIG1-LePRK/2 may be another part of the SPDC competing mode.

*In vitro*, LeSTIG1 competes with LAT52 to bind to the extracellular domain of LePRK2. The addition of LeSTIG1 to the mature pollen extract abolishes the interaction of LAT52 and LePRK2. The stigmatic signaling molecule LeSTIG1 (paracrine) competitively interacts with the extracellular domain of pollen-specific LePRK2, gradually displaces LAT52, and thereby switches from inhibiting pre-pollination germination to promoting pollen germination and tube elongation (Tang et al., 2004). Paracrine signaling competes with autocrine signaling, slow and gradually shutting down autocrine signaling to initiate a functionally opposite signaling pathway. We speculate that LeSTIG1 might have a similar or higher binding affinity for LePRK2 than LAT52, which requires microscale thermophoresis data for support.

Here, the LePRK1/2 receptor complex is a single pole, and pollen-specific LAT52 and stigma-specific LeSTIG1 are critical control switches. The switch of two ligands initiates and determines that the receptor complexes execute different or opposite functions. LAT52-LePRK1/2 inhibits pollen germination before maturation, while LeSTIG1-LePRK1/2 turns on pollen germination and tube growth after pollination, although their downstream components are not clear. Pollen monitors itself to inhibit germination until it receives the stigma-specific ligand signal to initiate germination and growth. The SPDC competing mode to inhibit or initiate pollen germination would be precise, stable, and efficient to ensure that fertilization is achieved.

## Papillary cells inhibit and induce pollen hydration

*Arabidopsis* stigma epidermal cells differentiate into protruding papillary cells, which are one of the few water-permeable sites in plants. The stigmatic papilla cells recognize compatible pollen that falls on them and transfer water to the dry pollen grains, which promotes pollen hydration and germination (Doucet et al., 2016; Dresselhaus et al., 2016). The molecular mechanism of inhibiting or promoting pollen hydration in stigma papilla cells can also be explained by the SPDC competing mode model. The membrane receptors FERONIA (FER)-ANJEA (ANJ) of papillary cells recognize the self-secreted Rapid Alkalization Factor (RALF) 23/33, and function to maintain a high level of reactive oxygen species (ROS) in papillary cells, and prevent water leakage (Liu et al., 2021). Once the pollen lands on the stigma, the pollen coat protein B-class (PCP-Bs) competitively binds to the FER-ANJ receptor complexes. This interrupts the ROS generation pathway activated by RALF23/33-FER/ANJ, initiates the PCP-Bs-FER/ANJ signaling pathway to reduce the ROS level, and triggers water release, which hydrates the pollen and promotes its germination. Thus, the FER-ANJ receptor complexes of papillary cells act as the “pole” in the SPDC competing mode. The RALF23/33 derived from papillary cells and the PCP-Bs secreted by pollen act as the dual control switches in the SPDC competing mode. RALF23/33 and PCP-Bs competitively interact with FER-ANJ to maintain or reduce ROS accumulation to prevent or provoke pollen hydration and germination (Liu et al., 2021).

ROS level are significantly reduced in papilla cells after pollination in *Arabidopsis* wild-type stigma (McInnis et al., 2006). ROS decreases at the sites of pollen adhesion and extends to adjacent areas along the edges of papilla cells. The application of ROS inhibitors and scavengers facilitates pollen hydration (McInnis et al., 2006; Liu et al., 2021). *Catharanthus roseus* receptor-like kinase 1-like (CrRLK1L) family members, ANJ and FER, are specifically expressed in the stigma at the stage11–14 during flowering, and persist until stigma maturation. In the *anj* and *fer-4* mutants, compared with the wild-type stigma, the ROS level is reduced, and the hydration rate of compatible pollen is accelerated; the phenotype is more evident in the *anj fer-4* double mutant (Liu et al., 2021). The LORELEI-like GPI-AP1 (LLG1) served as the co-receptor of FER-ANJ (Feng et al., 2019). The *llg1-2* mutant displayed a reduced ROS level in stigmatic papillae, similar to *fer-4* and *anj fer-4* mutants.

RALF23/33 are small cysteine-rich peptides. The *Arabidopsis ralf33* mutant has a phenotype similar to that of *anj* and *fer-4* mutants, with reduced ROS accumulation in the stigma and accelerated pollen hydration. Treatment with RALF33 induced increased ROS level in wild-type papilla cells without affecting ROS accumulation in the stigma of *anj-1 fer-4* mutant. RALF23/33 interacts with the extracellular domain of FER and ANJ in microscale thermophoresis and *in vitro* pull-down experiments (Liu et al., 2021). ANJ-FER has been thought to sense RALF33 and interact with ANJ-FER-LLG1 (Tsukamoto et al., 2010; Li et al., 2015), activating the downstream ROP2-RBOHD pathway to promote ROS generation (Duan et al., 2010; Kou et al., 2022), which maintains high ROS accumulation in un-pollinated papilla cells (Liu et al., 2021).

AtPCP-Bs are small cysteine-rich peptides and localized at pollen’s outer surface (Wang et al., 2017). Wild-type stigma pollinated with *pcp-bγ*, *pcp-bβ/γ*, or *pcp α/β/γ* mutant pollen displays obvious failure to reduce ROS accumulation, and pollen hydration slows (Wang et al., 2017; Liu et al., 2021). Wild-type stigma treated with PCP-Bγ shows a dose-dependent decrease in ROS level (Liu et al., 2021). *In vitro*, PCP-Bs compete with RALF33 to interact with the FER ectodomain in a dose-dependent manner (Wang et al., 2017). RALF33 interacts with the ectodomain of FER, with dissociation constants (*K*_d_) of 0.1604 μM. After adding PCP-Bγ to the mixture, PCP-Bγ competes with RALF33 to bind the FER ectodomain with an inhibition constant (*K*_i_) of 2.5099 μM in microscale thermophoresis assay (Liu et al., 2021).

*In vivo*, RALF33 treatment rescued the stigmatic papilla cells’ fast hydration phenotype in the *ralf33-3* mutant. While the addition of PCP-Bγ counteracted the effect of RALF33, and PCP-Bγ treatment restricts RALF33-induced stigmatic ROS generation in a dose-dependent manner. We suggested that PCP-Bγ competes with RALF33 to interact with the ectodomain of FER/ANJ, inhibiting RALF33-triggered stigmatic ROS production and promoting pollen hydration (Liu et al., 2021).

In summary, before pollination, the receptor complexes FER-ANJ in stigmatic papilla cells acts as a pole in the SPDC competing mode, receiving RALF23/33 secreted from papilla cells and interacting with co-receptor LLG1 (Tsukamoto et al., 2010; Li et al., 2015) to activate downstream ROS generation pathway of ROP2-RBOHD (Duan et al., 2010; Kou et al., 2022), and maintain ROS accumulation in papillary cells. The RALF23/33-FER/ANJ signaling pathway inhibits water release before papilla cells perceive pollen. When the compatible pollen arrives at the stigma, PCP-Bs, act as another switch molecule in the “dual control” mode, competing with RALF23/33 for interaction with the receptor complexes FER-ANJ, which reduces the RALF23/33-initiated signaling pathway, inhibits ROS production, and promotes pollen hydration (Liu et al., 2021). As female participants in pollen germination, stigma papilla cells maintain a dry stigmatic state through autocrine RALF23/33 signaling until they receive compatible pollen. PCP-Bs initiate the signaling that leads to pollen hydration and germination. The SPDC competing mode of pollen hydration is an exquisite, economical, simple, stable, and controllable mechanism in plant reproduction.

## Maintenance and disruption of pollen growth integrity

After pollen germination, the pollen tube transports two sperm cells as passive cargo, growing in the transmitting tract toward the ovule. During long-distance transportation, pollen tubes must maintain their integrity during growth. After reaching the destination, the pollen tube ruptures to release sperm cells. Plants thus require two mechanisms for maintaining the integrity during pollen tube growth and inducing the rupture of pollen tubes.

The SPDC competing mode perfectly addresses the opposite regulatory requirements. Here, pollen-specific Buddha’s Paper Sea 1/2 (BUPS1/2) (Ge et al., 2017) and ANXUR1/2 (ANX1/2) (Boisson-Dernier et al., 2009; Miyazaki et al., 2009) act as the “pole” of SPDC, and RALF4/19 derived from pollen tubes and RALF34 secreted by the inner integument serve as the “dual-control” switches of the SPDC competing mode. RALF4/19 regulates signaling through the receptor complex BUPS1/2-ANX1/2 to maintain integrity during pollen tube growth. RALF34 triggers another signaling pathway by competitively binding to the receptor complex BUPS1/2-ANX1/2 to induce pollen tube bursting. The dual control switches RALF4/19 and RALF34 regulate two diametrically opposite outcomes through competitively binding to the receptor complex BUPS1/2-ANX1/2.

BUPS1/2 and ANX1/2, members of the *Catharanthus roseus* RLK1-like family, are specifically localized at the tip of pollen tubes, forming BUPS1/2-ANX1/2 interaction complexes (Ge et al., 2017). *In vitro*, single, or double mutations of *BUPSs* and *ANXs* produce premature rupture phenotypes in pollen tubes but to different degrees (Boisson-Dernier et al., 2009; Miyazaki et al., 2009; Ge et al., 2017). *In vivo*, similar phenotypes also appeared in the wild-type stigma and style tract of plants pollinated with *bups1* or *bups1 bups2* pollen (Ge et al., 2017). BUPS1/2 and ANX1/2 are involved in maintaining the growth of pollen tubes and preventing pollen tubes from bursting in advance in order to release sperm cells at the right place.

The *RALF4/19* genes encode a 5-KD cysteine-rich rapid alkalizing factor that is expressed in mature pollen and pollen tubes. The double mutant *ralf4 ralf9* is male sterile (Morato do Canto et al., 2014). *In vitro*, the pollen of *ralf4 ralf9* germinated normally but bursted prematurely. *In vivo*, *ralf4 ralf9* pollen germination was normal but stagnated growth or ruptured directly on the stigma. It showed a premature rupture phenotype of pollen tubes similar to *bups1 bups2* double mutants.

*In vitro* experiments confirmed that BUPS1/2 and ANX1/2 can interact with RALF4/19 (Ge et al., 2017). Microscale electrophoresis assay showed that RALF4/19 interacts with the receptors BUPS1/2 and ANX1/2 with a similar affinity (Ge et al., 2017). As an autocrine signaling molecule, RALF4/19 interacts with the receptor complex BUPS1/2-ANX1/2 to coordinate the ROS signal generated by downstream NADH oxidase and regulated by MARIS, a receptor-like cytoplasmic kinase (RLCK) (Boisson-Dernier et al., 2015). This maintains the calcium ion gradient, regulates pollen tube growth, and maintains its integrity (Franck et al., 2018).

RALF34 is an ovule-specific small peptide. RALF34-GFP is specifically expressed in the ovule before fertilization, especially in the inner integument; it is secreted into the micropyle and around the synergid cells after fertilization. When applied *in vitro*, a low concentration of 2 nM RALF34 was sufficient to induce pollen tube rupture. RALF34 competes with RALF4/19 for interaction with BUPS1/2 and ANX1/2 (Ge et al., 2017). RALF34 may act as an ovule-driven paracrine signal to compete with autocrine RALF4/19, shutting down the signaling by RALF4/19 and resulting in pollen tube rupture and sperm cell release.

BUPSs and ANXs form heterodimers as “poles” in SPDC competing mode, mediating the precise spatiotemporal control of two antagonistic processes: pollen tube growth and rupture. Pollen tube-specific RALF4/19 and ovule-produced RALF34 are dual-switch molecules that act as distinct spatiotemporal triggers that mediate two opposite signal outputs: maintaining pollen tube growth integrity and triggering rupture. Signaling from the pollen (autocrine) peptide maintains tube growth integrity during the long journey to the ovule; an exchange ligand from the female tissue (paracrine) disarms the integrity of the pollen tube growth and initiates rupture to release sperm cells. A series of iterative developmental events in the correct sequence ensures successful plant reproduction. The SPDC competing mode controlling this iterative development process is accurate and efficient.

## Antagonistic peptides fine-tune stomatal patterning.

*Arabidopsis* stomatal development occurs through specific cell state transition events. First, a protoderm meristemoid mother cell (MMC) carries out the asymmetric division to produce a meristemoid, which asymmetrically divides and renews itself, creating surrounding stomatal lineage ground cells (SLGCs). Transcription factors SPEECHLESS (SPCH) and SCREAM1/2 (SCRM1/2) determine the initiation and proliferation of stomatal development. Second, the meristemoid loses its stem cell-like characteristics and differentiates into a guard mother cell (GMC), regulated by MUTE and SCRM1/2. Third, the GMC divides symmetrically to form a pair of guard cells which will surround the future pore when stomatal differentiation is complete, as determined by FAMA and SCRM1/SCRM2 (Lau and Bergmann, 2012; Pillitteri and Torii, 2012; Han and Torii, 2016). The entire development of stomata is programmed sequentially. Thus far, evidence suggests that the first two steps of the process are regulated by two sets of peptide–receptor kinase complexes in SPDC competing mode.

Firstly, an SPDC competing mode directly regulates the inhibition and initiation of asymmetric division of MMCs. ERECTA (ER) and TOO MANY MOUTH (TMM) receptor complexes act as the “pole” of the SPDC competing mode, and EPIDERMAL PATTERNING FACTOR2 (EPF2) and STOMAGEN function as dual control switches. EPF2 is secreted by epidermal cells and blocks initiation of the stomatal cell lineage through the interaction with ER-TMM on the epidermal cell membrane. STOMAGEN is secreted by mesophyll cells and diffuses to the outer surface of epidermal cells. STOMAGEN competes with EPF2 to interact with ER-TMM complexes, which initiates the asymmetric division of MMC, and produces SLGCs. EPF2 and STOMAGEN, as the dual control switches of SPDC competing mode, prevent or trigger the asymmetric division of MMC through the ER-TMM receptor complexes and govern the opposite outcomes of this signaling pathway.

EPF2, the secreted cysteine-rich peptide, is a negative regulator of stomatal development. The *EPF2* gene is expressed in epidermal cells early in stomatal development and the SLGCs is increased in the *epf2* mutant. In treatment with the bioactive EPF2 peptide, the epidermis of the leaf was composed only of pavement cells, with the same phenotype as the *spch* mutant (Lampard et al., 2008; Lee et al., 2012).

ER, a member of the ER family of cell surface leucine-rich repeat receptor-like kinases, assisted by a co-receptor, the receptor-like protein TMM, senses EPF2 to limit the initiation of stomatal cell lineages. The stomatal distribution is clustered in the *er erl1 erl2* and *tmm* mutants (Geisler et al., 2000; Shpak et al., 2005). During stomatal cell lineage initiation, the EPF2 peptide is perceived by ER-TMM, activating downstream intracellular components mediated by a mitogen-activated protein kinase (MAP kinase) cascade that directly phosphorylates and downregulates SPCH to inhibit the division of MMC (Lampard et al., 2009; Lee et al., 2012; Lee et al., 2015). The *EPF2* gene is the direct target of SPCH and SCRM. The EPF2, ER-TMM, and SPCH-SCRM modules form a negative feedback loop that generates a spatial pattern of uniform distribution of MMCs in stomatal development (Lau et al., 2014; Horst et al., 2015).

STOMAGEN/EPFL9 is a positive regulator of stomatal development (Kondo et al., 2010). Reduced *EPF9* expression produced a pavement-only phenotype similar to the *spch* mutant or wild-type treated with EPF2. STOMAGEN initiates asymmetric division of the MMC (Hunt and Gray, 2009; Sugano et al., 2010). Both STOMAGEN and EPF2 can bind to the extracellular domains of ER and TMM with similar dissociation constants *(K*_d_). STOMAGEN and EPF2 directly compete for binding to the same receptor, ER, with a maximum inhibitory concentration of 454 nM. EPF2 applied to wild-type seedlings inhibited stomatal development. Then, treatment with STOMAGEN rescued this phenotype by increasing STOMAGEN concentrations to counteract the excess EPF2, thus promoting stomatal differentiation (Lee et al., 2015).

Thus STOMAGEN and its antagonistic peptide EPF2 competitively binding to the ER-TMM to orchestrate the decision to initiate stomatal development and give rise to the stomatal cell lineage. Binding of STOMAGEN to ER blocks the function of EPF2 and closes the EPF2-ER/TMM-SCRM/SPCH feedback signaling pathway. The stomatal MMC enters asymmetric division, initiates proliferation, and generates SLGCs (Lee et al., 2015). EPF2 and STOMAGEN, the dual controller switch of SPDC competing mode, competitively bind to the ER-TMM receptor complexes, finely coordinating the MMC division at the appropriate spatiotemporal window.

Secondly, the SPDC competing mode orchestrates the inhibition and initiation of the GMCs. Similarly, the ERECTA-LIKE1 (ERL1) and TMM receptor complexes on the epidermal cell membrane serve as the “pole” of SPDC competing mode, and EPIDERMAL PATTERNING FACTOR1 (EPF1) derived from epidermal cells and STOMAGEN secreted by mesophyll cells serve as dual control switches in SPDC competing mode. EPF1, via its interaction with ERL1-TMM on the membrane, inhibits the differentiation of the meristemoid. STOMAGEN accumulates on the outer surface of epidermal cells and interacts with ERL1-TMM to unlock the GMC formation. EPF1 and STOMAGEN, as the dual-control switches of SPDC competing mode, block or unlock the formation of GMCs by competitively binding the ERL1-TMM receptor complexes to coordinate two antagonistic signaling pathways.

EPF1 is a negative regulator of stomatal development, whose coding gene is expressed during the transition from the meristemoid to the GMC. The *epf1* mutant has an abnormal spacing phenotype, suggesting it has a role in stomatal spacing and differentiation. EPF1 primarily interacts with ERL1, an ER family member, to regulate stomatal spacing and prevent meristemoid differentiation and GMC formation.

ERL1 is induced by SPCH in early stomatal lineage cells and expressed in stomatal cells, early meristems, and SLGCs, but strongly accumulates during the transition from meristemoid to GMC (Lau et al., 2014; Qi et al., 2017). EPF1-ERL1 signaling is activated with the assistance of the co-receptor TMM to target and downregulate MUTE expression, which controls the differentiation of the meristemoid. Overlapping expression of EPF1, ERL1, and MUTE triggers the downregulation of MUTE in the narrow developmental window of the late meristemoid-to-GMC transition, preventing GMC formation (Qi et al., 2017).

STOMAGEN/EPF9 proteins, diffusing into the pro-epidermal meristemoid, compete with EPF1 for direct binding to ERL1/TMM and shut down the EPF1-ERL1/TMM-SCRM/MUTE signaling pathway that inhibits meristemoid differentiation, provoking meristemoids to differentiate into GMCs (Lee et al., 2015; Qi et al., 2017).

Two sets of cognate ligand–receptor pairs, EPF2-ERECTA and EPF1-ERL1, act on the core stomatal transcription factors SPCH and MUTE, respectively, to limit stomatal development. However, the regulation of SPCH and MUTE differs in functional frameworks. The meristemoid block is caused by the initiation of ERL1 signaling activated by endogenous EPF1. However, EPF2 is required to suppress the stomatal developmental potential in epidermal cells. STOMAGEN might counteract the repression of stomatal development, which is engaged by EPF2-ER, by competing with EPF2 for binding to the ER. STOMAGEN turns off the inhibitory effect of EPF1-ERL1/TMM-MUTE autocrine signaling by competing with EPF1 for binding to ERL1 and initiating meristemoid differentiation into GMC. The receptor complexes ER-TMM and ERL-TMM are the pole in the SPDC competing mode. EPF2/EPF1 and STOMAGEN act as antagonistic switch signals and implement dual control by competing to interact with the receptor complexes (pole).

## Switch between mechanisms of symbiosis and immunity

During long-term evolution, plants utilize different strategies against microbial infection, establish symbiotic and immune relationships, and achieve adaptability. Plants defend against pathogens to provoke pathogen-associated molecular pattern (PAMP)-triggered immunity (PTI) and effector-triggered immunity (ETI); symbiotic fungi inhibit PTI and ETI to establish symbiosis with plants (Liu et al., 2007; Yu et al., 2021). During symbiosis between legumes and *Rhizobia*, root nodules exhibit a weaker defense response to *Rhizobia* (Benezech et al., 2020). In non-legumes, adding Nod factors causes the suppression of PTI immunity triggered by flg22 (Liang et al., 2013). The coordination mechanism of establishing symbiosis or immunity between plants and symbiotic fungi can be perfectly interpreted with the SPDC competing mode. Low molecular weight chitotetraose (CO4) and chitohexaose (CO8) are products of chitin decomposition. CO4 and CO8 are the double control switches in the SPDC competing mode, and the receptor kinase OsCERK1 (CHITIN ELICITOR RECEPTOR KINASE 1) is the pole. CO8 derived from pathogenic fungi activates MAPKs cascade to trigger PTI and establish immunity through the OsCERK1-OsCEBiP (CHITIN ELICITOR-BINDING PROTEIN) receptor complexes. CO4 derived from mycorrhizal symbiotic fungi inhibits CO8-activated MAPKs defense and establishes symbiosis via OsCERK1-OsMYR1 (MYC FACTOR RECEPTOR 1). CO4 and CO8 compete for the receptor kinase OsCERK1 to control two antagonistic signal pathways.

Chitin is a significant component of the cell walls of symbiotic and pathogenic fungi. It acts as a microbial molecule-associated pattern (MAMP) that triggers PTI (Yu et al., 2021). Short-chain chitooligosaccharides (COs) and nonsulfated lipochitooligosaccharides secreted by Arbuscular mycorrhizal (AM) fungi serve as signal molecules to mediate symbiosis (Maillet et al., 2011; Zhang et al., 2021). Short-chain or long-chain chitooligosaccharides are from AM fungi or pathogenic fungi (Feng et al., 2019), inducing plants to inhibit or promote defense responses and establish symbiosis or immunity, respectively (Liang et al., 2013; Sun et al., 2015).

The receptor-like kinase OsCERK1 with a lysine motif (LysM) recognizes symbiosis and immune signals in rice. The CO4 of AM fungi can recruit the co-receptor OsMYR1 to interact with OsCERK1 (Xin et al., 2012). The pathogenic fungal chitosan oligosaccharide CO8 can induce the heterodimerization of OsCEBiP and OsCERK1 (Shimizu et al., 2010). Whether OsCERK1 initiates symbiotic or immune pathways depends on which ligand it interacts with, the CO4 or CO8 ligand. When the short-length chitosan oligosaccharide ligand CO4 of AM fungi is recognized by OsCERK1, CO4 and the co-receptor OsMYR1 occupy OsCERK1, which inhibits the interaction between OsCERK1 and the immune co-receptor OsCEBiP, lessening the phosphorylated level of OsGEF1 by OsCERK1 to decrease the sensitivity to PAMP (Yamaguchi et al., 2013). When OsCERK1 senses CO8 from pathogenic fungi, CO8 promotes the co-receptor OsCEBiP to interact with OsCERK1, competes with OsMYR1, and takes over OsCERK1, blocks the formation of the CO4-OsMYR1-OsCERK1 complexes, promotes immunity, and suppresses symbiosis. The *OsCEBiP* disruption mutant showed a higher rate of mycorrhizal colonization at the early stage of infection. OsMYR1-overexpressed transgenic plants are more sensitive to fungal pathogens (Zhang et al., 2021).

OsCERK1, as the pole in the SPDC competing mode, senses trigger CO4 and forms the CO4-OsMYR1-OsCERK1 complexes, which weakens defense responses and promotes symbiosis. OsCERK1 recognizes elicitor CO8, which forms the immune complex CO8-OsCEBiP-OsCERK1, and competes to block the formation of the symbiotic complexes CO4-OsMYR1-OsCERK1, establishing an immune mechanism (Zhang et al., 2021). The switch molecule “CO8” or “CO4” and the control receptor “OsCERK1” form different ligand–receptor complexes to form the two antagonistic signaling pathways of immunity or symbiosis.

## Discussion

Both SPDC and SPDT in the circuit regulate two opposite or exclusive pathways. The difference is that the SPDT switch in the circuit is mainly controlled by the “pole” to determine which circuit is connected. In the process of signaling initiation, the controller is that the different ligands compete for the receptor and receptor complexes. The ligand–receptor interaction determines which signaling pathway is initiated, so ligand competition governs which signaling pathway is activated. Receptors or receptor complexes acting as “poles” are passive in terms of which pathway is activated.

The mode of two ligands competing for receptor complexes could not be explained by the competition model of enzyme–substrate–inhibitor. In the competitive inhibition model, the structure of the inhibitor is similar to that of the substrate, occupying the active center of the enzyme, and preventing the substrate from entering the active center to achieve the purpose of inhibition. Paracrine ligands are structurally similar to autocrine ligands, compete to bind to the receptor complexes and inhibit the interaction between autocrine ligands and receptor complexes. In this respect, paracrine ligands have the same effect as inhibitors. However, the difference is that the interaction between the paracrine ligand and receptor complex turns off the signal pathway initiated by the autocrine ligand and triggers another signal pathway. Therefore, we could not simply use the competitive inhibition model to explain the SPDC competing mode.

Moreover, if the paracrine and autocrine ligands do not belong to the same family, they may have different binding sites on the receptor, resulting in changes in the receptor’s conformation to turn off the signal pathway initiated by autocrine ligands and switch on the signal pathway initiated by paracrine ligands. The competitive inhibition model would not be suitable to explain the molecular mechanism of this type of interaction. More experimental data is needed to reveal the molecular basis of how ligands competitively bind to receptors (Yu et al., 2022).

Paracrine signal molecules are secreted and diffuse, accumulating sufficient concentrations to interact with receptor complexes competitively. Whether or not paracrine peptides have a higher binding affinity for receptor complexes than autocrine peptides? Really, STOMAGEN and EPF2 interact with the extracellular domain of ER with dissociation constants (*K_d_*) of 44 nM and 59 nM, respectively (Lee et al., 2015). STOMAGEN appeared to have a slightly greater binding affinity to the ER ectodomain than EPF2. On the contrary, PCP-Bs and RALF33 interacted with the ectodomain of FER, with dissociation constants (*K_d_*) of 0.34 μM and 0.1604 μM, respectively (Liu et al., 2021). RALF33 (autocrine peptide) has a greater binding affinity to the FER ectodomain. PCP-Bs might be accumulated to a slightly higher concentration for competing with RALF33 to interact with FER-ANJ. If paracrine and autocrine peptides interact with receptor complexes with similar dissociation constants. Then, paracrine signal molecules compete with autocrine ones but do not entirely replace them. Only with an increase in concentration does the paracrine signal gradually replace the autocrine signal to interact with the receptor complexes. when the concentration of paracrine peptides is high enough locally, it may completely substitute for autocrine peptides.

In plants, the surface receptor kinases receive extracellular signals, activate downstream factors, establish regulation modules by signaling molecule–receptor kinase complexes–downstream cascade reaction elements, and regulate gene expression. Cells respond immediately to developmental cues, hormone levels, and microbial invasion. Generally, a single activated or inhibited signaling pathway is established. We call this the “single-pole single-control” mode. For example, TPD1 (TAPETUM DETERMINANT 1) and EMS1(EXCESS MICROSPOROCYTES1)/EXS (EXTRA SPOROGENOUS CELLS) play essential functions in anther cell fate determination (Jia et al., 2008). Due to various receptors, there is a group of multifaceted receptors undergo “single-pole” and “single-control” mode in different cells. For example, FERONIA receives RALF17/23 to regulate the immune processes (Stegmann et al., 2017), perceives RALF1 to control root development (Yu et al., 2020), and even recognizes RALF6/7/16/36/37 during double fertilization to trigger polytubey block (Zhong et al., 2022). In these examples, “single-pole single-control” regulates a single signaling pathway or outcome. However, the “SPDC” competing mode orchestrates a sequence of developmental processes via antagonistic peptides that act as dual competitive controllers.

In an SPDC competing mode, a set of receptors or receptor complexes can perceive two or two sets of ligand signals which competitively controls two different or even antagonistic signaling pathways. Different ligands act as switches to initiate the function of the receptor complexes. The SPDC competing mode is the most straightforward and economic mechanism for executing complex and variable regulation purposes, simplifying plant genomes, endowing plants with optimal functions, and achieving optimal biological adaptability.

Autocrine signals, which activate receptor complexes, maintain the state of a cell and inhibit entry into a subsequent developmental program. Under suitable conditions, a paracrine signal will compete with and gradually substitute for the autocrine signal bound to receptor complexes, weakening the signaling pathway orchestrated by the autocrine signal, and initiating the antagonistic process or initiating the subsequent program. An SPDC competitive regulation mode would maintain appropriate development with error correction and prevention functions. It might be a simple and efficient protection mechanism produced in the process of plant evolution to ensure the programmed progress of growth and development. In plants, more iterative developmental events may be precisely orchestrated by an SPDC competing mode. It is still a mystery to be explored and discovered.

Multicellular organisms orchestrate cell proliferation, expansion, and differentiation to achieve ultimate pattern formation. In terrestrial plants, the long-distance transport of water and nutrients is completed by the xylem and phloem, which are developed and differentiated from vascular stem cells (procambium). PXY (PHLOEM INTERCALATED WITH XYLEM), CLV1(CLAVATA 1), and BAM1/2/3 (BARELY ANY MERISTEM 1/2/3), belong to the LRR-RLK XI subfamily (Fisher and Turner, 2007). PXY is specifically expressed in procambial cells to maintain their stem cell properties. In the *pxy* mutant, the procambial layer disappeared and failed to form space intervals between the phloem and the xylem. The xylem was inserted into the phloem regions, and the order of cell arrangement was destroyed (Fisher and Turner, 2007). It was speculated that PXY might maintain the procambium’s stem cell characteristics and promote the xylem’s differentiation. TDIF (tracheary element differentiation inhibitory factor) is the twelve peptides (HEVHYPSGSHYPISN; HYP, 4-hydroxyl acid) secreted from the phloem (Hirakawa et al., 2008). TDIF interacts with PXY to promote the procambium’s polarization and proliferation to inhibit differentiation of vascular stem cells into the xylem. TDIF, the paracrine signal secreted by the phloem, binds to the PXY receptor in the procambium and promotes cell division of the procambium and differentiation of the xylem. We predict an autocrine ligand would be present in the procambium to maintain stem cell characteristics, however there is currently no experimental evidence for this. According to our SPDC competing mode, there might be a ligand that acts to maintain stem cell characteristics and inhibition of procambial cell division.

Additionally, in the regulation of the inflorescence architecture, EPFL4/6 (EPIDERMAL PATTERNING FACTOR 4/6) peptide signals derived from the endodermis, a layer adjacent to the phloem (Abrash et al., 2011; Uchida et al., 2012; Uchida and Tasaka, 2013) are perceived by ER, a receptor, in the pholem. It is unclear whether there might be an autocrine signal to monitor the self-development status of the phloem. How the EPFL4/6 paracrine signal is recognized to initiate inflorescence development remains a mystery. According to the SPDC competing mode, the autocrine signal would be found, which competes with EFL4/6 to bind to the ER receptor in the cell surface of the phloem.

Immunity and symbiosis are antagonistic relationships between plants and microbes. During the microbial invasion, plants adopt the SPDC competing mode in selecting symbiosis and immunity. We have a slightly different view from Zhang et al. (Zhang et al., 2021) who propose that OsCERK1 acts as a single-pole; the co-receptor and ligand complex, CO8-OsCEBiP and CO4-OsMYR1, acted as dual-controlled switch molecules, which then interact with OsCERK1 to establish immune or symbiotic relationships. We suggest that ligands act as dual-control switch molecules to organize their respective signaling complexes and activate the signaling pathways. In the adaptive relationship of immunity and symbiosis between plants and microbes, the signals in the SPDC competing mode come from microbes. They trigger distinct signaling pathways by organizing different receptor complexes to establish symbiosis or immunity. We believe that this would allow for a more adaptive mechanism for interactions between plants and microbes.

## Conclusion

We put forth evidence that there are three narrow developmental windows during the plant’s reproduction process are controlled by a SPDC competing mode. Pollen maintains the LAT52-LePRK1/2 signaling pathway, which inhibits its germination until it arrives at the stigma and receives the LeSTIG1 signal secreted by the pistil. LeSTIG1 competes with LAT52 to interact with LePRK1/2, which weakens the LAT52-LePRK1/2 signaling pathway, and initiates LeSTIG1-LePRK1/2 signaling to facilitate pollen germination. However, in stigma papilla cells, RALF23/33-FER/ANJ-LLG1 induces ROS production and inhibits water release until the arrival of compatible pollen. The pollen-coated proteins PCP-Bs compete and gradually replace RALF23/33 for interaction with FER/ANJ, reducing ROS generation and accumulation, and promoting hydration. Finally, during the long journey to the ovule, the RALF4/19-BUPSs/ANXs-LLGs signaling pathway maintains the growth and integrity of the pollen tubes until it reaches the ovules, and perceives the RALF34 secreted by the inner integument. RALF34 competes with RALF4/19 to interact with the BUPSs/ANXs-LLGs receptor complexes, triggering the rupture of pollen tubes and releasing sperm cells.

In the development of stomata, two regulation modules are consistent with the SPDC competing mode. In epidermal cells, EPF2-ER-TMM inhibits the initiation and proliferation of stomatal lineage cells until receiving STOMAGEN secreted from mesophyll cells, which competes with EPF2 to interact with ER, removing the inhibition caused by EPF2-ER-TMM, and promoting MMC development. Similarly, EPF1-ERL1-TMM inhibits meristemoid development and differentiation into GMCs until STOMAGEN competes with EPF1, binding to ERL1-TMM, relieving meristemoid inhibition, and initiating its differentiation.

**Figure 2:**
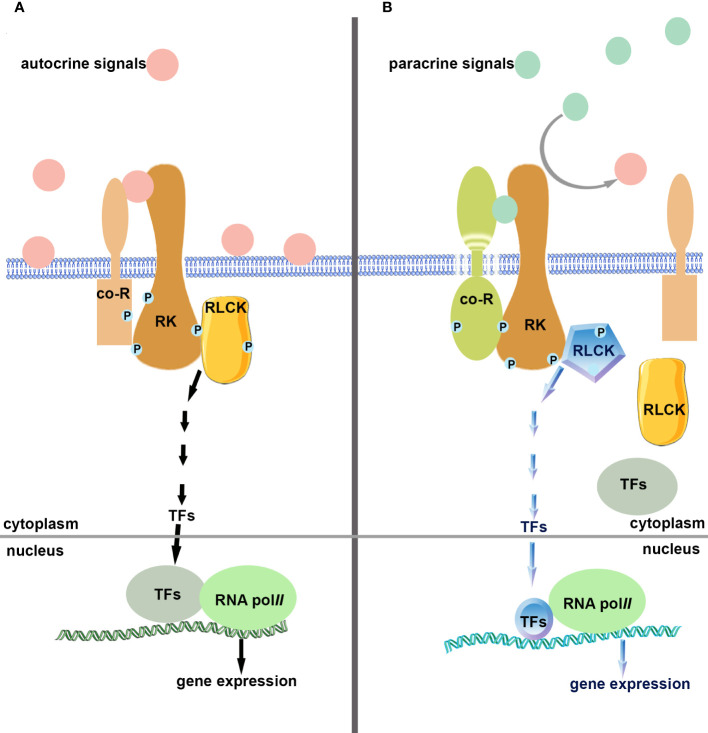
Schematic diagram of single-pole dual-control competing mode. **(A)**. Autocrine signals (ligands) bind and activate receptor kinase (single-pole) and their co-receptors, activate intracellular components, regulate gene expression, monitor cells, and prevent premature carrying out of the following developmental processes. **(B)**. Paracrine signals (ligands) compete and bind to receptor kinases (single-pole) and their co-receptors, shutting down autocrine signaling pathways, activating downstream elements, regulating gene expression, and turning on signaling pathways that differ from autocrine ones.

According to temporal or location information, sequential iterative developmental events can be orchestrated in an SPDC competing mode in a narrow spatiotemporal window during plant development (as shown in **Figure 2**). It might be a universal mechanism. The SPDC competing mode in plant development and microbial adaptation could act as an adaptative mechanism produced by natural selection during plant evolution. Whether SPDC competing modes widely exist in other signal transduction processes remains to be explored through more literature review and empirical investigation.

## Author contributions

T-YG, W-JL, and D-LC gathered literature and participated in discussions. T-YY designed the projects, wrote this paper, and prepared Figures. All authors reviewed the manuscript. All authors contributed to the article and approved the submitted version.
